# *In vivo *antinociception of potent mu opioid agonist tetrapeptide analogues and comparison with a compact opioid agonist - neurokinin 1 receptor antagonist chimera

**DOI:** 10.1186/1756-6606-5-4

**Published:** 2012-01-30

**Authors:** Karel Guillemyn, Patrycja Kleczkowska, Alexandre Novoa, Bart Vandormael, Isabelle Van den Eynde, Piotr Kosson, Muhammad Faheem Asim, Peter W Schiller, Mariana Spetea, Andrzej W Lipkowski, Dirk Tourwé, Steven Ballet

**Affiliations:** 1 Department of Organic Chemistry, Vrije Universiteit Brussel, Pleinlaan 2, B-1050 Brussels, Belgium; 2 Mossakowski Medical Research Centre, Polish Academy of Sciences, Pawinskiego street 5, 02106 Warsaw, Poland; 3 Department of Pharmaceutical Chemistry, Institute of Pharmacy and Center for Molecular Biosciences Innsbruck, University of Innsbruck, Innrain 52a, A-6020 Innsbruck, Austria; 4 Department of Chemical Biology and Peptide Research, Clinical Research Institute of Montreal, 110 Avenue des Pins Ouest, Montreal, Canada

**Keywords:** Opioid tetrapeptides, dual opioid agonist - neurokinin antagonist peptidomimetic, tolerance studies

## Abstract

**Background:**

An important limiting factor in the development of centrally acting pharmaceuticals is the blood-brain barrier (BBB). Transport of therapeutic peptides through this highly protective physiological barrier remains a challenge for peptide drug delivery into the central nervous system (CNS). Because the most common strategy to treat moderate to severe pain consists of the activation of opioid receptors in the brain, the development of active opioid peptide analogues as potential analgesics requires compounds with a high resistance to enzymatic degradation and an ability to cross the BBB.

**Results:**

Herein we report that tetrapeptide analogues of the type H-Dmt^1^-Xxx^2^-Yyy^3^-Gly^4^-NH_2 _are transported into the brain after intravenous and subcutaneous administration and are able to activate the μ- and δ opioid receptors more efficiently and over longer periods of time than morphine. Using the hot water tail flick test as the animal model for antinociception, a comparison in potency is presented between a side chain conformationally constrained analogue containing the benzazepine ring (BVD03, Yyy^3^: Aba), and a "ring opened" analogue (BVD02, Yyy^3^: Phe). The results show that in addition to the increased lipophilicity through amide bond N-methylation, the conformational constraint introduced at the level of the Phe^3 ^side chain causes a prolonged antinociception. Further replacement of NMe-D-Ala^2 ^by D-Arg^2 ^in the tetrapeptide sequence led to an improved potency as demonstrated by a higher and maintained antinociception for AN81 (Xxx^2^: D-Arg) vs. BVD03 (Xxx^2^: NMe-D-Ala). A daily injection of the studied opioid ligands over a time period of 5 days did however result in a substantial decrease in antinociception on the fifth day of the experiment. The compact opioid agonist - NK1 antagonist hybrid SBCHM01 could not circumvent opioid induced tolerance.

**Conclusions:**

We demonstrated that the introduction of a conformational constraint has an important impact on opioid receptor activation and subsequent antinociception in vivo. Further amino acid substitution allowed to identify AN81 as an opioid ligand able to access the CNS and induce antinociception at very low doses (0.1 mg/kg) over a time period up to 7 hours. However, tolerance became apparent after repetitive i.v. administration of the investigated tetrapeptides. This side effect was also observed with the dual opioid agonist-NK1 receptor antagonist SBCHM01.

## Background

Opioids are the most widely used analgesics in the treatment of various pain states. Early binding studies and functional bioassays defined three main types of opioid receptors in the central nervous system: μ- (MOR), δ- (DOR) and κ- (KOR) receptors [1,2]. The μ-opioid receptor was identified to be essential for an efficient antinociception in acute and severe pain models. Aside from the desired pain-relieving action, prolonged exposure to μ opioids results in well established, undesired side effects, including sedation, respiratory depression, physical dependence and analgesic tolerance [3,4]. Several studies however provided evidence that compounds with a dual MOR/DOR activity present beneficial pharmacological effects in comparison to highly selective MOR agonists [5]. It was, for example, demonstrated that the analgesic efficacy of MOR agonists can be enhanced by DOR agonists [6,7]. Moreover, mixed MOR agonism/DOR (ant)agonism can suppress or eliminate the development of physical dependence, tolerance and respiratory depression, adverse effects which are typically observed for selective MOR agonists [5,8,9]. Because of these promising indications, compounds which combine MOR agonism with DOR (ant)agonism have received a great deal of attention as lead structures towards novel opioid analgesics.

A limitation of most peptides for use as analgesics consists of their limited access to the brain. This drawback is not only due to their poor metabolic stability, but also a consequence of their low lipid solubility and limited ability to cross the blood-brain barrier (BBB) [10]. Several strategies have been developed to overcome the hurdle of opioid peptide drug delivery to the brain, including structural modifications such as glycosylation [10,11], lipid conjugation [12], and various types of cyclization [13-15]. In this way researchers attempt to modulate the balance of hydrophobic-hydrophilic properties of the compounds and to decrease the number of accessible conformations, which can potentially result in an increased membrane permeability. Despite the preparation and evaluation of a plethora of bioactive opioid ligands, both peptidic and non-peptidic, an alternative to morphine, the golden standard in moderate to severe pain research, has yet to be found.

An alternative to the preparation of compounds which solely target the opioid system consists of the creation of hybrid compounds that interact with multiple biologically relevant targets [16-18]. The emerging strategy to design and synthesize chimeras, also called designed multiple ligands (DMLs), as a promising alternative to a treatment by monotherapy, is justified by the fact that such compounds are commonly characterized by not only a high activity, but more importantly present the possibility to improve the pharmacological profile by selectively 'designing out' an undesired biological characteristic, both in terms of action or transport [19,20]. As such, the chemical structure of the drug allows to modulate the permeability of the active substance and in consequence create "site specificity of action" [9,16]. The precision of action of these kinds of compounds can be built up by the knowledge about their specific activity as single molecules. By hybridizing two active compounds into one chemical entity it is possible to develop drugs which may provide a superior delivery capacity, as compared to the delivery of a physical mixture of the drugs.

When applied to pain research the combination of an opioid pharmacophore and a NK1 receptor antagonist led to a new type of bifunctional drugs which aim at a prolonged therapeutic efficiency over time [21-24]. Substance P, an 11-amino acid peptide belonging to the tachykinin family, is known to be important for transmission of nociceptive signals [25]. The design rationale of the opioid-NK1 DMLs originates from the upregulation of the pronociceptive neurotransmitter substance P and its receptor, the neurokinin 1 receptor (NK1R), upon chronic administration of opioids [26,27]. The NK1 antagonist pharmacophore in these DMLs could potentially counter the enhanced expression of substance P and its preferential receptor, NK1R.

**Figure 1:**
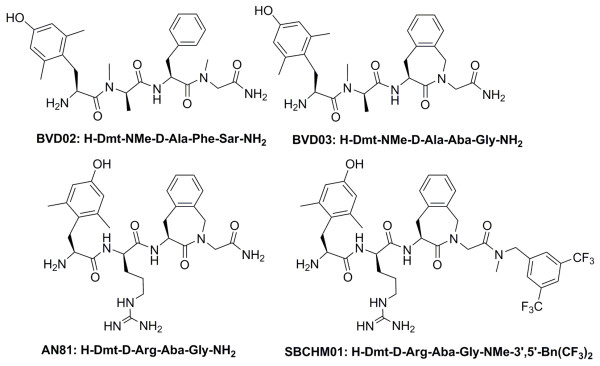
**Structures of opioid ligands BVD02, BVD03, AN81 and opioid-NK1 chimera SBCHM01**.

From our previous results, we selected three opioid tetrapeptides with promising *in vitro *potency for further *in vivo *evaluation. All three reported peptide analogues were derived from dermorphin (H-Tyr-D-Ala-Phe-Gly-Tyr-Pro-Ser-NH_2_) as this exogenous peptide proved to have a high affinity and selectivity for the MOR [28,29]. Additionally, its use as a lead structure was also motivated by the fact that dermorphin, when administered centrally or peripherally in rodents, induces less tolerance development when compared to morphine [30]. We recently identified the opioid ligands H-Dmt-NMe-D-Ala-Phe-Sar-NH_2 _(BVD02) [31], H-Dmt-NMe-D-Ala-Aba-Gly-NH_2 _(BVD03 with Aba: 4-amino-1,2,4,5-tetrahydro-2-benzazepin-3-one, see Figure 1) [31] and H-Dmt-D-Arg-Aba-Gly-NH_2 _(AN81) [32] to be potent compounds *in vitro *(Figure 1 and Table 1). The N-terminal Tyr^1 ^present in dermorphin was replaced by 2',6'-dimethyl-(*S*)-tyrosine (Dmt) to improve the enzymatic stability, receptor affinity and opioid potency [33], whereas a conformationally constrained aminobenzazepinone (Aba) moiety placed in third position maintained MOR affinity as well as activation and resulted in a favorable increase in DOR binding and activity [34-36].

**Table 1:** *In vitro *functional activities and affinities of opioid- and opioid-NK1 ligands

Compound	NK1R pA_2_^a^	*h*NK1R K_i _(nM)^b^	MOR EC_50 _(nM)^c, d^	DOR EC_50 _(nM)^c, d^	MOR K_i_ (nM)^e, f^	DOR K_i_ (nM)^e, f^	KOR *K_i_* *(nM)^e, f^*
**BVD02**	/	/	0.079 ± 0.0065^c^	4400 ± 500^c^	60 ± 3^e^	130 ± 5^e^	> 10^6 e^
**BVD03**	/	/	0.00174 ± 0.00034^c^	0.016 ± 0.009^c^	15 ± 2^e^	5 ± 3^e^	> 10^6 e^
**AN81**	*/*	*/*	0.32 ± 0.04^d^	0.42 ± 0.02^d^	0.15 ± 0.02^f^	0.60 ± 0.07^f^	118 ± 12^f^
**SBCHM01**	7.8	0.5 ± 0.1	8.51 ± 0.62^d^	43.3 ± 6.3^d^	0.416 ± 0.012^f^	10.4 ± 0.6^f^	445 ± 81^f^

^a ^The pA_2 _value was calculated using the Schild's equation [47]. ^b ^Inhibitory constants (K_i_) of NK1 receptor ligands, measured for the receptor prototype [^3^H]-SP in the presence of hNK1-CHO membranes. Results are means ± SEM of three independent experiments. Binding data were calculated using the nonlinear regression/one site competition fitting options of the GraphPad Prism Software. ^c ^Agonist properties of peptides on forskolin-stimulated cAMP accumulation by MOR and DOR [31,48]. ^d ^Values represent means of 3-6 experiments ± SEM in the GPI (functional assay is representative of MOR activation) and MVD (DOR-representative assay) tissue bioassays [32,49]. ^e ^Binding affinities of compounds for MOR and DOR were determined by displacing [^3^H]diprenorphine in HEK293 cells stably expressing MOR, DOR and KOR [31]. ^f ^Displacement of [^3^H]DAMGO and [^3^H]DSLET, respectively, from rat brain membrane binding sites and binding affinities for κ opioid receptors were measured by displacement of [^3^H]U69,593 from guinea pig brain membrane binding sites [32].

The Aba benzazepine moiety was also used as a central scaffold in the design of a compact bifunctional ligand, SBCHM01, which possessed the desired dual opioid agonist - NK1R antagonist activity *in vitro *(Figure 1 and Table 1) [32].

Herein, we describe an *in vivo *evaluation of these peptide derivatives after peripheral (intravenous i.v. and subcutaneous s.c.) administration in mice in order to verify their ability to cross the BBB and produce centrally induced analgesia. In addition, the analgesic potency of a compact hybrid opioid agonist - NK1R antagonist ligand (SBCHM01) was determined to investigate the influence of the NK1 pharmacophore incorporated at the C-terminus of the opioid subunit. The propensity of all four peptides to induce tolerance after repetitive administration in mice is reported.

## Results

### *In vivo *antinociception of the opioid tetrapeptides BVD02, BVD03, AN81 and the hybrid opioid agonist - NK1R antagonist SBCHM01

The antinociceptive potency of three opioid tetrapeptides H-Dmt-NMe-D-Ala-Phe-Sar-NH_2 _(BVD02), H-Dmt-NMe-D-Ala-Aba-Gly-NH_2 _(BVD03) [31] and H-Dmt-D-Arg-Aba-Gly-NH_2 _(AN81) [32], and of the hybrid opioid-NK1 structure H-Dmt-D-Arg-Aba-Gly-NMe-3',5'-Bn(CF_3_)_2 _(SBCHM01) was investigated after peripheral administration. Following a previously reported procedure [37], each compound was administered intravenously into mice and the hot water tail-flick test was used to measure the analgesic effect induced by these compounds.

**Figure 2:**
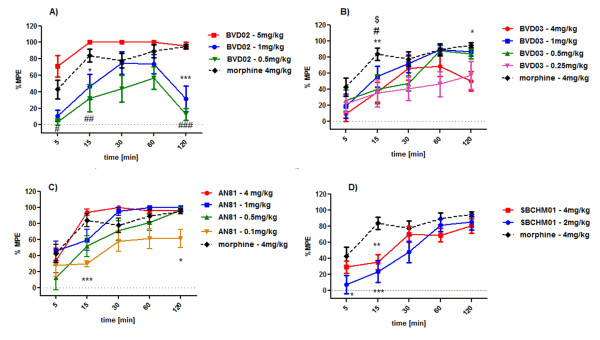
**The dose- and time-dependent analgesic activity of A) BVD02, B) BVD03, C) AN81 and D) SBCHM01 after i.v. injection at two to four different doses and in comparison with morphine (4 mg/kg, i.p.) Each compound was tested using C57Bl6 male mice and hot water tail-flick test was used**. Data are represented as the % of maximal possible effect (%MPE) ± SEM of 6-10 mice. Statistics conducted using a two-way analysis of variance (ANOVA) followed by Bonferroni's test showed significant differences (*p < 0.05; **p < 0.01; ***p < 0.001) between the examined peptides and morphine. Differences are defined as: * for BVD02 (1 mg/kg) and # for BVD02 (0.5 mg/kg); * for BVD03 (4 mg/kg); # BVD03 (1 mg/kg); $ BVD03 (0.5 mg/kg) and * for AN81 (0.1 mg/kg).

The time- (F_(4;23) _= 24.41) [38,39] and dose-dependent (F_(3;23) _= 6.006) curves of analgesic response induced by the BVD02 opioid tetrapeptide, are presented in Figure 2A. BVD02 injected at a dose of 5 mg/kg produces a strong analgesic effect, which reaches the maximum level at the early time point of 15 min post-injection (%MPE = 100) and remains unchanged within the next hour. Only at 2 h after its administration a slight decrease in activity is observed (%MPE = 96 ± 4; p > 0.05), thus producing a similar analgesic effect as morphine (4 mg/kg) (%MPE = 95.08 ± 1.67). In contrast, BVD02 at doses of 1 and 0.5 mg/kg displays noticeably lower antinociceptive activity. The highest BVD02-mediated antinociception is reached at 30 min (1 mg/kg; %MPE = 74.77 ± 13.63; p > 0.05) and at 60 min (0.5 mg/kg; %MPE = 56.77 ± 13.57; p > 0.05), respectively. At the next time point (120 min post-injection) a dramatic reduction of activity is observed (p < 0.001) when compared to morphine.

BVD03 was administered into mice at four different doses, ranging from 0.25 mg/kg to 4 mg/kg, and produced time- (F_(4;32) _= 33.15) and dose-dependent (F_(4;32) _= 3.166) antinociceptive activity as depicted in Figure 2B. Interestingly, analgesia induced by this compound injected at a dose of 4 mg/kg seems to be weaker than that induced by morphine at the same dose (4 mg/kg). The highest analgesic activity of BVD03 (4 mg/kg) is observed at 1 h post-injection (%MPE = 68.78 ± 7.18; p > 0.05). The following measurement at 120 min after drug injection results in significant reduction of analgesia, when compared to morphine (%MPE = 50.06 ± 6.84; p < 0.05). The subsequently investigated doses of BVD03 (1 mg/kg and 0.5 mg/kg) produced comparable antinociceptive responses at 60 min and 120 min post-injection. The calculated percentage of the maximal possible effect for the concentration of 1 mg/kg is: %MPE = 89.21 ± 5.22 (60 min; p > 0.05) and %MPE = 86.69 ± 5.52 (120 min; p > 0.05), whereas for BVD03 administered at 0.5 mg/kg the MPE values are 88.41 ± 7.15 (60 min; p > 0.05) and 84.29 ± 6.46 (120 min; p > 0.05), respectively.

A decrease in analgesic response of BVD03 injected into mice is observed at a dose of 0.25 mg/kg. Remarkably, comparing to doses of 4 and 0.5 mg/kg, a similar antinociceptive effect (%MPE = 35-40%) induced at 15 min post-injection is seen. However, at further time points dose-response curves are characterized by having distinct profiles. In case of BVD03 (0.25 mg/kg) a gradual growing profile, reaching the maximum level of %MPE = 57.48 ± 17.57 at 2 h post-injection, is recorded.

In all cases, except at a dose of 0.25 mg/kg, the conformationally constrained peptide analogue BVD03 shows a drop in antinociception after 2 hours. Given this observation it was decided that additional assays over longer time periods were redundant.

The analogous tetrapeptide analogue AN81, H-Dmt-D-Arg-Aba-Gly-NH_2_, differs from BVD03 by only one amino acid (i.e. D-Arg^2 ^vs. NMe-D-Ala^2^, resp.), and proved to possess a superagonist profile after *in vitro *evaluation [32]. In the functional GPI and MVD tissue bioassays, the measured subnanomolar EC_50 _values were 0.32 nM and 0.42 nM and corresponding subnanomolar binding affinity was observed (K_i_μ 0.15 nM and K_i_δ 0.60 nM).

In contrast to BVD03, AN81 induces very consistent high antinociceptive responses even at low doses as shown in Figure 2C. When administered at a dose of 4 mg/kg, the opioid ligand AN81 exhibits an effect comparable to the effect induced by the same compound at a 4-fold lower dose (1 mg/kg). At the 30 min post-injection time point the %MPE values for both 4 and 1 mg/kg doses are %MPE = 100 (p > 0.05) and %MPE = 95 ± 2.89 (p > 0.05), respectively. Interestingly, a two-way ANOVA followed by Bonferroni's post-hoc test, shows no difference in the analgesic effect between first three drug doses examined doses (4 mg/kg, 1 mg/kg and 0.5 mg/kg) and morphine at all investigated time points (p > 0.05). In contrast, this compound induced a significantly lower analgesic effect, with respect to intraperitoneally (i.p.) administered morphine (4 mg/kg), at a low dose of 0.1 mg/kg at 15 min and 120 min after injection. However, the percentage of maximal possible effect exceeds 50% reaching the value %MPE = 61.2 ± 6.45 (p < 0.05) even after 2 h which illustrates the long duration of action of this compound.

**Figure 3:**
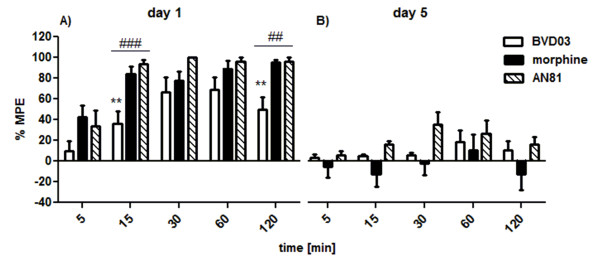
**Analgesic effect induced by chronic intravenous injection of AN81 and BVD03 opioid peptides at a dose of 4 mg/kg**. The examined drugs were administered daily for five days (between 10 and 12 am). The determination of nociceptive responses was done on the first (Figure 3A) and fifth day (Figure 3B) of the experiment. %MPE ± SEM of 7-10 animals per group. On the first day of measurements BVD03, but not AN81, show significant differences (**p < 0.01) when compared to morphine. Additionally, # represents differences which occur between examined compounds compared to each other. In contrast, on the fifth day statistics do not show any significant differences (*p < 0.05; **p < 0.01;***p < 0.001).

Next, both opioid tetrapeptide ligands BVD03 and AN81 were also examined with regard to their propensity to induce tolerance development (Figure 3). For this purpose, each compound was injected intravenously at a dose of 4 mg/kg and a 5 day-long chronic treatment was carried out.

The systemic and repeated administration (a daily regimen for 5 days) of both compounds leads to the development of tolerance (Figure 3B). On the first day of drug application both BVD03 and AN81 induce strong antinociceptive responses. BVD03 is again characterized as being a weaker analgesic than AN81. While AN81 develops almost constant analgesia, which varies between 94 and 100% of MPE starting from 15 min post-injection until the last time point (2 h), the increase in antinociceptive activity of BVD03 halts 1 h post-injection, reaching a %MPE = 68.78 ± 7.18 (Figure 3A). On the fifth day of the experiment the maximum level of AN81-mediated analgesia is reached 30 min post-injection where it still shows a %MPE = 34.99 ± 7.32 (p < 0.001), whereas with BVD03 only a low MPE value of %MPE = 18.14 ± 6.84 was determined after 60 min. At subsequent time points the pain-relieving action of AN81 is gradually reduced, achieving at the last time point the MPE value of 15.83% ± 4.40 (p < 0.001).

**Figure 4:**
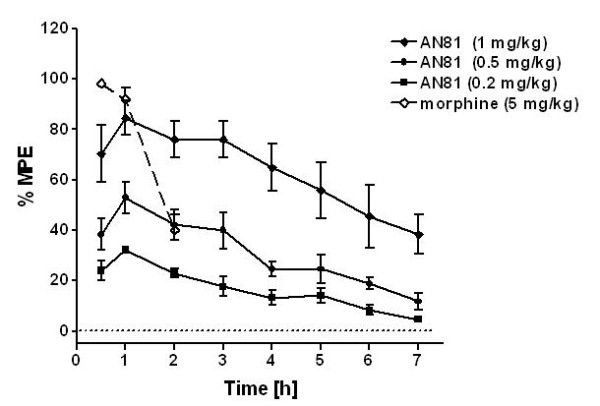
**Dose and time dependent analgesic effects of AN81 after s.c. administration in the radiant heat tail-flick test**. AN81 or morphine was injected s.c. into CD1 mice. Data are %MPE ± SEM of 5-6 mice.

In order to further investigate the duration of action of AN81, the radiant heat tail-flick test was used, which allowed the measurement of the analgesic response over a longer time period. The antinociceptive effects of AN81 were measured up to 7 h after s.c. administration in mice. (Figure 4).

The antinociceptive activity of AN81 was determined after s.c. administration of 0.2, 0.5 and 1 mg/kg. As shown in Figure 4, AN81 produced dose-dependent effects with a peak of antinociception action at 1 h after administration at all three doses. After this time point and within further time points a slow but constant decrease in activity is observed. It is however remarkable that an antinociceptive effect is measured up to seven hours after s.c administration of AN81 at a dose of 1 mg/kg (%MPE = 38.4 ± 7.8%). This stands in steep contrast to morphine for which the activity drops rapidly over time.

Next, we focused on the pharmacological role that an additional NK1R pharmacophore could have in these opioid tetrapeptide ligands. After establishment of the potent *in vitro *antinociceptive effect of SBCHM01 [32] an analgesic animal assay *in vivo *would provide information on: i) whether or not this type of chimeric compound is actually able to cross the BBB, ii) any potentiation/synergy of the analgesic effect due to the NK1R antagonist subunit being present in this chimeric molecule and iii) if tolerance can be suppressed or even eliminated by the presented hybrid approach, as suggested in the literature [40,41].

Figure 2D shows a significant analgesic effect after administration of the hybrid peptide SBCHM01 at two different doses. As it can be observed that i.v. administration of SBCHM01 at a dose of 2 mg/kg produces a similar antinociceptive response as compared to a dose of 4 mg/kg. This observation is most prominent in the later time points, i.e. 1 h and 2 h post-injection. Moreover, at the last time point of the measurement, the %MPE for both examined doses as well as for morphine (4 mg/kg) is at the same level and varies between 80.47 ± 5.29 (4 mg/kg), 85.12 ± 5.43 (2 mg/kg) and 95.08 ± 1.67 (morphine, 4 mg/kg).

**Figure 5:**
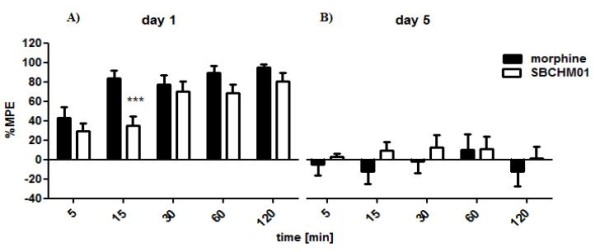
**SBCHM01-induced tolerance after systemic administration**. SBCHM01 was injected i.v. at a dose of 4 mg/kg daily for 5 days. The results scored as %MPE were compared with morphine (i.p.) at the same dose. **(A) **The SBCHM01-mediated antinociception measured on the first day of the study; **(B) **Reduction of SBCHM01 analgesic activity on the fifth day of the experiment. Each column represents mean ± SEM of 8-10 mice. ***p < 0.001 significantly different from morphine-injected animals.

**Figure 6:**
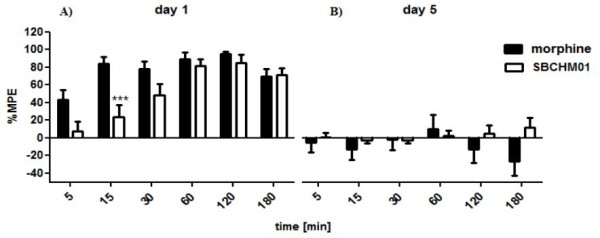
**Comparison of a time-response profile of SBCHM01 (2 mg/kg, i.v.) and morphine (4 mg/kg, i.p.) after chronic administration into mice and prolonging the experiment time up to 3 h**. **A) **SBCHM01-mediated analgesia on the first day of measurements; **B) **Complete abolishment of SBCHM01's antinociceptive activity on the fifth day of the experiment.

After comparison with the above mentioned opioid-induced tolerance of BVD03 and AN81 (Figure 3), we observed similar results after chronic administration of SBCHM01 (Figure 5 and 6). As can be seen from these graphs, an injection of this chimera on the first day at a dose of 4 mg/kg induces a strong time-dependent (F_(4;17) _= 13.81) analgesic response (Figure 2D, 5A). However, when compared with i.p. administered morphine given at the same dose, SBCHM01 seems to be slightly weaker during the whole period of measurements. This also correlates with the reported *in vitro *data which indicated that, relative to AN81, SBCHM01 possessed a reduced *in vitro *activity in both the GPI and MVD assay (Table 1). At 2 h post-injection, the %MPE values of 80.47 ± 5.29 (p > 0.05) and 95.08% ± 1.67 are reached for SBCHM01 and morphine, respectively.

Subsequently, a daily injection of both morphine and SBCHM01 was carried out over a 5-day period in order to verify whether these compounds are prone to the development of tolerance. Unfortunately, on the fifth day of the experiment mice had developed opioid-induced tolerance (Figure 5B), only weak analgesia still being observed (time-point of 30 min; %MPE = 12.25 ± 7.33).

The repetitive administration of SBCHM01 was also performed using a 2-fold lower dose (2 mg/kg) and an additional measurement at 180 min post-injection was carried out. At that time point a decrease in potency is clearly visible for both SBCHM01 and morphine (Figure 6A).

As can be seen from Figure 6, lowering the i.v. bolus dose from 4 to 2 mg/kg does not alter the antinociceptive activity significantly (cfr. Figure 5A) as it still remains high on the first day of treatment (Figure 6A). Again, a gradual build-up of SBCHM01-mediated analgesia is seen up to 120 min post-injection (%MPE = 85.12 ± 5.43). However, on the first day of the experiment, 3 h after drug administration, a moderate reduction of analgesia is observed (%MPE = 71.13 ± 4.71). Additionally, from the acquired data we could conclude that a lower dose of SBCHM01 does not reduce the extent of tolerance development (Figure 6B).

## Discussion

The potent antinociceptive effects of the investigated structures after peripheral administration indicate that all four peptidic compounds are able to cross the BBB. The antinociceptive enhancement concomitant with the introduction of a conformational constraint at the level of the Phe^3 ^residue (BVD02 vs. BVD03) indicates that next to molecular lipophilicity, an additional factor for efficient analgesia in these peptidic ligands consists of the limitation in conformational flexibility. The observed effects are in agreement with earlier *in vitro *binding data obtained for these peptides (BVD02→BVD03: K_i_μ 60 → 15 nM, K_i_δ 130 → 5 nM, Table 1) [31], which also confirm that the conformational restriction imposed by the Aba moiety maintains the MOR binding affinity of the ligands, but substantially ameliorates DOR binding. The receptor affinities were in agreement with functional *in vitro *MOR and DOR activity, as determined by a functional forskolin-stimulated cAMP accumulation assay (BVD02→BVD03: EC_50_μ 0.079 → 0.00174 nM, EC_50_δ 4400 → 0.016 nM, Table 1) [31]. The aforementioned role of delta receptors in the modulation of efficacy of MOR agonists needs to be considered at this point. Next to the MOR activation needed for efficacious analgesia, the superior MOR binding and potency of BVD03 in comparison to BVD02 (factor of 4 and 45, respectively), is accompanied by a 26-fold increase in DOR affinity and a 10^4 ^gain in DOR potency. Given the proposed synergy of mixed MOR agonists-DOR agonists in analgesia, it is likely that both MOR and DOR components of BVD03 are responsible for the enhanced *in vivo *antinociception when compared to BVD02. The activity data suggest that next to the N-methylation in the "ring opened" BVD02, leading to an increased lipophilicity and potentially higher bioavailability, an additional constraint of the Phe side chain by use of Aba in BVD03 is also beneficial for antinociception (Figure 2A and 2B).

In an earlier study, and based on the peptide sequence of the lead MOR agonist Dmt^1^-DALDA (H-Dmt-D-Arg-Phe-Lys-NH_2_) [42], which is a potent opioid analgesic [18], we prepared and evaluated the *in vitro *pharmacological opioid profile of H-Dmt-D-Arg-Aba-Gly-NH_2 _(AN81)_. _This compound was also identified as a full MOR/DOR agonist with subnanomolar affinity and tissue bioactivity (Table 1) [32]. The structure differs from BVD03 only in the amide nitrogen N-methyl group and side chain of the second residue in the peptide sequence (Figure 1). When an activity comparison is made between the two peptides at different doses (see Figure 2B and 2C), AN81 seems to produce a faster build-up in antinociceptive response over time at a dose of 4 mg/kg, which also seems to be maintained during the first two hours of measurements. From Figure 3, we concluded that at that dose of 4 mg/kg AN81 is superior to BVD03 and furthermore possesses a very high %MPE value even after two hours. AN81's remarkable antinociceptive potency is observed at doses ranging from 4 mg/kg (i.v.) to 0.5 mg/kg (i.v.) (Figure 2C), showing that by lowering the dose there is the possibility to obtain the same long-lasting pain-relieving effect. To have a better idea of the total duration of action of the best tetrapeptide analogue (AN81), an additional *in vivo *pain test was used. We switched to the radiant tail-flick assay to avoid the infliction of a possible tail injury after repetitive measurements in the hot-water test. Such an injury could in turn result in the erroneous measurements of antinociception as observed in earlier studies by our group upon chronic administration of potent drugs.

A subcutaneous (s.c.) administration of AN81 at three different doses (1, 0.5 and 0.2 mg/kg) induced marked analgesia lasting up to 7 hours, whereas morphine lost almost all activity over a time period of 2 hours at more elevated doses, relative to AN81 (Figure 4). Because of the *in vivo *potency and long duration of action, these results place AN81 on the list of potentially promising active lead compounds for acute pain treatment.

The ability of the two most active compounds AN81 and BVD03 to circumvent one of morphine's major and highly problematic side effects, the development of tolerance upon chronic opioid administration was subsequently tested. Figure 3 clearly demonstrates that a daily dose of both peptide ligands generate an important reduction in antinociception of the fifth day of the experiment. Only the most active derivative, AN81, showed a partially preserved activity with a maximum (ca. 35%MPE) at the time point of 30 min post-injection. An observation which can potentially be correlated to the balanced MOR/DOR agonism of AN81.

The combination of an opioid pharmacophore and a NK1 receptor antagonist in one chemical entity has been identified as an approach towards a new type of bifunctional drugs which aim at a prolonged therapeutic efficiency over time [21-24]. When a 3',5'-bistrifluoromethyl benzyl moiety is coupled to the C-terminus of AN81, a compound with dual opioid - NK1 *in vitro *activity results (Table 1) [32]. After systemic injection, SBCHM01 produced a significant dose- and time-dependent analgesia during 2 to 3 hours (Figure 5 and 6, resp.). The inferior antinociceptive effect of this chimera, when compared to its 'pure' opioid analogue AN81 (Figure 2C and 2D), could not only be ascribed to a diminished MOR affinity and activation (Table 1), but is also in agreement with the large decrease in DOR binding and potency (17- and 103-fold, resp.). The results obtained for SBCHM01 indicate that neither an synergistic nor potentiating antinociceptive effect appears when both pharmacophores are combined in a single chemical entity.

The opioid-induced development of tolerance observed with BVD03 and AN81 (Figure 3), was in this study unfortunately not reduced by the additional presence of a NK1R antagonist pharmacophoric group covalently attached to the C-terminus of the opioid tetrapeptide AN81 (Figures 5 and 6). Concomitant with the substantial loss of DOR agonism in SBCHM01, the beneficial property of dual MOR/DOR agonists in lowering opioid-induced tolerance is unfortunately eliminated, and could explain the complete loss in antinociception on the fifth day of the experiment. This in contrast to the partial preservation of analgesia observed after repetitive administration of AN81, a balanced and potent MOR/DOR agonist. (Figure 3).

Recently, Vanderah and coworkers [43] reported the detailed *in vivo *evaluation of H-Tyr-D-Ala-Gly-Phe-Met-Pro-Leu-Trp-O-3',5'-Bn(CF_3_)_2 _(TY005) in several animal assays to define the role of this compound in thermal and tactile stimuli in uninjured, sham- and nerve-injured male, Sprague-Dawley rats. They observed that this compound was able to exert the desired dual activity *in vivo*. The opioid agonist activity and NK1R antagonism were demonstrated in independent assays in which one of the two activities was isolated by blocking the second functionality. The authors showed that the development of antihyperalgesic tolerance could be suppressed by this multimodal ligand. This compound was further optimized by Hruby and coworkers by introducing a Dmt residue in position 1 and replacing the metabolically labile ester function by an amide group and demonstrated that these changes resulted in improved opioid agonism, while maintaining NK1 activity [24].

Upon comparison of this study with related work by other research teams [43,44], it is however important to point out that different animal models and conditions (animals, assay type, drug delivery) were applied. In the recent publication of Largent-Milnes *et al. *[43] the inhibition of antihyperalgesic tolerance with the hybrid opioid-NK1 TY005, was demonstrated after i.t. administration and by use of the paw withdrawal test. This is in contrast to the current study, where the hot water tail-flick assay served as a model for acute pain and in which the drug was injected intravenously. The various types of noxious stimuli employed in the experimental paradigms, as well as the different administration routes, make it difficult to rationalize the different mechanisms that are responsible of this apparent discrepancy.

## Conclusions

In the present study AN81 (H-Dmt-D-Arg-Aba-Gly-NH_2_) exhibits the strongest analgesic effect, when administered systemically, which suggests that this peptidomimetic has the capacity to penetrate the highly selective blood brain barrier. Moreover, this opioid tetrapeptide analogue is characterized by a long duration of action after subcutaneous injection and proved to be more active than a very similar structure with a NMe-D-Ala residue in the second position of the sequence, i.e. BVD03. This conformationally restricted peptide ligand proved in turn to be more potent that its "ring opened" analogue BVD02.

Unfortunately, AN81 appeared to still induce the development of tolerance after repetitive administration over a time period of five days when tested in the hot water tail-flick test. When compared to morphine, the induced tolerance was however markedly lower. A similar result was obtained for the bifunctional opioid agonist-NK1R antagonist ligand, SBCHM01. This peptidic chimera was, in analogy with the 'pure' opioid ligands, capable of reaching and activating opioid receptors in the CNS, but it lost its antinociceptive potency completely upon chronic injection as determined in the hot water tail-flick assay.

The apparent inability of SBCHM01 to suppress tolerance development appears to oppose recent reports by Vanderah and coworkers [43] which indicate that a hybrid opioid-NK1 octapeptide ligand (TY005) was able to attenuate tolerance development related to sustained opioid pathophysiology in an hyperalgesic model after central (i.t.) administration, as opposed to this study in which i.v. administration was used in an acute pain model. For a general clinical applicability, an important goal in pain research consists of the discovery and development of improved analgesic drugs which could be administered systemically. Hence, we are convinced that unravelling the cause of this intriguing discrepancy is important and will consequently be the target of our future SAR studies.

## Methods

### In vivo measurement of drug-induced analgesia

#### A) Intravenous drug administration

Nociceptive responses to thermally-induced pain were assessed by the hot water tail-flick test [45] using male C_57_Bl_6 _mice. Animals (weighing 25-28 g) were maintained on a normal light-dark cycle and testing occurred during the light cycle. Mice were injected intravenously (i.v.) with the examined drug, dissolved in saline, at different doses. For the verification of drug-induced antinociceptive tolerance, the investigated compounds were administered (i.v.) once per day for 5 days (2 and 4 mg/kg for SBCHM01 and 4 mg/kg for both AN81 and BVD03) and the analgesic effect was measured on the first and the fifth day of the experiment. In each case (both acute and chronic treatment) the effect was measured over a minimum time period of 120 minutes. The antinociceptive effect was measured in triplicate at the following time points post-injection: 5, 15, 30, 60, 120 min (and 180 min for SBCHM01; due to the strong and long-lasting analgesic effect produced by AN81 (Figure 2C), there was no possibility to prolong the experiment, which - if prolonged - could in consequence cause major damage to the animal's tail, thus giving spurious nociceptive responses). Responses to thermal hot stimulus were assessed by the latency with which the mouse removed its tail from 55°C water. A cut-off time of 10 s was used in order to prevent tissue damage.

The antinociceptive activity exhibited by all analogues were determined as a percentage of the maximal possible effect (%MPE) calculated as: %MPE = [(posttreatment latency - baseline latency)/(cut-off latency - baseline latency)] × 100. In order to compare the antinociceptive effect induced by the examined drugs, morphine was used as a standard reference.

All experimental procedures used in this animal testing followed the guidelines on ethical standards for the investigation of experimental pain in animals and were approved by the Animal Research Committees of the Medical Research Centre, Polish Academy of Sciences.

#### B) Subcutaneus drug administration

In order to determine the duration of drug activity, s.c. drug administration into male CD1 mice (27-33 g; Charles River Laboratories, Sulzfeld, Germany) was performed. In addition to a thermal nociceptive stimulus, the alternative light beam-based tail-flick assay [46] was performed using an UB 37360 Ugo Basile analgesiometer (Ugo Basile s.r.l., Varese, Italy). The reaction time required by the mouse to remove its tail due to the radiant heat was measured and defined as the tail-flick latency. A cut-off time of 10 s was used in order to minimize tissue damage. Tail-flick latencies were measured before (baseline) and after drug s.c. administration (30 min, 1 h, 2 h, 3 h, 4 h, 5 h, 6 h and 7 h), the antinociceptive effect was scored as a percentage of the maximal possible effect (% MPE), according to formula presented above. Drugs were dissolved in sterile physiological saline (0.9%) and were administered s.c. in a volume of 10 μl per 1 g body weight. The animal procedures were approved by the Austrian Ethical Committee on Animal Care and Use in line with international laws and policies.

### Statistical analysis

Data are presented as means ± standard errors of the mean (SEM). All of the calculations and statistical analysis were performed using Prism 5.0 (Graph Pad Software, San Diego, CA, USA). The nociceptive scores were analyzed using a two-way Anova with Bonferroni's post hoc test. The p value (the value of probability) as well as inferential statistics - F test [38,39], which include information about the obtained magnitude or value of the test statistic and degrees of freedom, are represented.

## List of abbreviations

Aba: 4-amino-1,2,4,5-tetrahydro-2-benzazepin-3-on; BBB: blood-brain barrier; CNS: central nervous system; DML: designed multiple ligand; Dmt: 2',6'-dimethyl-(*S*)-tyrosine; DOR: δ-opioid receptor; GPI: Guinea pig ileum; KOR: κ-opioid receptor; MOR: μ-opioid receptor; MPE: maximal possible effect; MVD: mouse vas deferens; NK1: neurokinin 1.

## Competing interests

The authors declare that they have no competing interests.

## Authors' contributions

All authors contributed equally in the research and preparation of the manuscript. All authors approved the final manuscript.
